# Research on Enhanced Dynamic Pig Counting Based on YOLOv8n and Deep SORT

**DOI:** 10.3390/s25092680

**Published:** 2025-04-24

**Authors:** Peng Shen, Keyu Mei, Haori Xue, Tenglong Li, Guoqing Zhang, Yongxiang Zhao, Wei Luo, Liang Mao

**Affiliations:** 1 North China Institute of Aerospace Engineering, School of Aeronautics and Astronautics, Langfang 065000, China; shen01111@aliyun.com (P.S.); 18130679197@163.com (K.M.); xhr5120@163.com (H.X.); 15512897981@163.com (T.L.); 2 North China Institute of Aerospace Engineering, School of Remote Sensing and Information Engineering, Langfang 065000, China; zhanggq@stumail.nciae.edu.cn (G.Z.); zhaoyx@stumail.nciae.edu.cn (Y.Z.); luowei@radi.ac.cn (W.L.); 3 Guangdong-Hong Kong-Macao Greater Bay Area Artificial Intelligence Application Technology Research Institute, Shenzhen Polytechnic University, Shenzhen 518055, China

**Keywords:** pig counting, object detection, multi-object tracking, YOLOv8n, Deep SORT

## Abstract

Pig counting is an essential activity in the administration of pig farming. Currently, manual counting is inefficient, costly, and unsuitable for systematic analysis. However, research on dynamic pig counting encounters challenges, including inadequate detection accuracy stemming from crowding, occlusion, deformation, and low-light conditions. Target tracking issues characterized by poor accuracy, frequent identity confusion, and false positive trajectories ultimately lead to diminished accuracy in the final counting outcomes. Given these existing limitations, this paper proposes an enhanced algorithm based on the YOLOv8n+Deep SORT model. The ELA attention mechanism, GSConv, and VoVGSCSP lightweight convolution modules are introduced in YOLOv8n, which improve detection accuracy and speed for pig target recognition. Additionally, Deep SORT is enhanced by integrating the DenseNet feature extraction network and CIoU matching algorithm, improving the accuracy and stability of target tracking. Experimental results indicate that the improved Deep SORT-P pig tracking algorithm attains MOTA and MOTP values of 89.2% and 90.4%, respectively, reflecting improvements of 4.2% and 1.7%, while IDSW is diminished by 25.5%. Finally, counting experiments were performed on videos of pigs traversing the farm passage using both the original and improved algorithms. The improved YOLOv8n-EGV+Deep SORT-P algorithm achieved a counting accuracy of 92.1%, reflecting a 17.5% improvement over the original algorithm. Meanwhile, the improved algorithm presented in this study successfully attained stable dynamic pig counting in practical environments, offering valuable data and references for research on dynamic pig counting.

## 1. Introduction

In the daily farming and management of large-scale farms, it is essential to conduct regular inventory and counting of livestock. Standardized and refined agricultural practices are progressively becoming the norm to enhance farming efficiency [1]. By utilizing pig counting data to regulate feed quantity and type, and formulating customized farming strategies for various breeds and sizes of pigs, the profitability of pig farming can be significantly enhanced.

In traditional livestock farms, common methods for counting pigs include manual counting and the use of wearable sensors [2]. However, with the continuous expansion of farming scale, manual counting has become increasingly impractical, placing a significant burden on the physical and mental resources of farm workers. Moreover, if the staff is inattentive or fatigued, manual counting is prone to substantial errors. The use of wearable sensors, such as RFID ear tags, also faces considerable challenges. First, tagging each pig individually in large herds is a labor-intensive task and often induces stress responses in the animals. Additionally, these electronic tags may cause a certain degree of physical discomfort or injury to the pigs. Over time, the tags may become detached due to pig behaviors such as biting, rubbing, or fighting, leading to inaccuracies in counting and increased operational costs. Furthermore, in large-scale farms, especially in facilities with dense populations and frequent occlusions, sensor-based systems often suffer from signal interference or recognition failures, particularly in pigpens with extensive metal structures. In view of these limitations, this study adopts a fixed-camera-based approach, integrating object detection and tracking techniques for pig counting and monitoring. Compared with traditional manual counting and sensor-based methods, vision-based approaches offer a higher degree of automation and intelligence, significantly reducing labor and operational costs. More importantly, they allow for accurate pig identification and counting even in complex environments, without causing any harm or discomfort to the animals.

Driven by the rapid development of artificial intelligence and big data, deep learning (DL) algorithms, characterized by multi-layer neural networks, have achieved notable breakthroughs across diverse domains, including image classification, computer vision, and time series classification [3,4]. DL algorithms have been extensively utilized in the field of object detection owing to their superior accuracy and rapidity [5,6,7]. These algorithms can be classified into two categories: two-stage detection methods and single-stage detection methods. Two-stage detection methodologies encompass Region-based Convolutional Neural Networks (RCNN) [8], Fast Convolutional Neural Networks (Fast-RCNN) [9], and Mask Convolutional Neural Networks (Mask-RCNN) [10], which exhibit superior detection accuracy. Single-stage detection methods include SSD [11], YOLOv3 [12], and others, which offer advantages in processing speed. All of these deep learning algorithms have demonstrated great accuracy in object detection and localization, rendering them reliable and consistent models for animal monitoring.

Numerous researchers have investigated animal detection and identification systems. Hansen et al. [13] applied facial recognition technology to pigs, extracting biometric features for non-invasive detection and identification. Song et al. [14] proposed a YOLOv3-p model for sheep facial detection by optimizing anchor box dimensions and reducing model parameters, which improved detection accuracy while decreasing computational complexity. Yang et al. [15] created a lightweight sheep face detection model based on Retina Face, employing a streamlined backbone network to minimize model parameters and optimizing the network’s loss function, achieving a detection accuracy of 97.1%. For behavioral analysis, Chae et al. [16] identified cattle mating behavior using an enhanced object detection network. By incorporating additional convolutional layers and sampling layers into YOLOv3, they proposed a four-scale detection network with 98.5% accuracy. For livestock counting applications, Yang et al. [17] enhanced the YOLOv5n algorithm for pig counting by constructing a multi-scene pig dataset and integrating the SE channel attention module. This approach enhanced both the accuracy and robustness in complex occlusion scenarios, achieving a mean absolute error (MAE) of 0.173. Similarly, Hao et al. [18] introduced an improved YOLOv5-based pig detection and counting model, which integrates the shuffle attention mechanism and Focal-CIoU loss, achieving a mean average precision (mAP) of 93.8% for detection and 95.6% for counting. In recent years, numerous computer vision algorithms have been progressively applied to animal husbandry. Free-roaming animal counting systems for cattle [19,20], sheep [21], and wildlife [22] assist researchers and herders document behavioral patterns and activity zones. Huang et al. [23] modified the SSD detection network with InceptionV4 modules to enhance the detection accuracy of cattle tails in movement corridors, integrating augmented Kalman filter and Hungarian algorithm for tracking. Cao et al. [24] achieved dynamic counting of a limited number of sheep by integrating the improved YOLOv5x and Deep SORT algorithms, supplemented by the ECA mechanism, while maintaining a low error rate.

Some scholars have made significant progress in pig counting algorithms. Cowton et al. [25] combined Faster R-CNN for detection with Deep SORT for tracking and implemented a convolutional network-based re-identification algorithm to track pigs in sties. The tracking data were utilized to measure total distance traveled, idle time, and average speed. Stavrakaki et al. [26] developed a Kinect motion detection system using reflective markers on pigs’ necks to transmit movement data to receivers positioned along the channel, distinguishing healthy from lame pigs by analyzing locomotion sounds. For direct counting applications, Tian et al. [1] converted RGB images into density distribution maps and employed an enhanced CNN network to accurately count 15 pigs. Kim et al. [27] implemented pig counting on the NVIDIA Jetson Nano embedded platform (NVIDIA, Santa Clara, CA, USA), installing cameras above the pigsty corridors to record pigs’ movements. They utilized the lightweight model Tiny YOLOv4 for detection and introduced a lightweight tracking method, LightSort, by enhancing the re-identification module of Deep SORT. A line-crossing approach was employed to enumerate the pigs. Chen et al. [28] proposed a real-time automated counting system with a single fisheye camera, achieving accurate counts through bottom-up pig detection, deep convolutional neural networks for keypoint recognition and association, online tracking, and a novel spatiotemporal response filtering mechanism. Huang et al. [29] enhanced pig counting accuracy by improving YOLOv5x with embedded dual-sized SPP networks and replacing MaxPool operations with SoftPool, integrating this with Deep SORT for tracking. A comprehensive review of the literature reveals persistent challenges in pig counting: mutual occlusion and overlapping among pigs, target loss and frequent identification switching during tracking, and false positive tracking trajectories. Although counting algorithms share basic architectural similarities, significant variations exist in camera angles, lighting conditions, pig appearance features, and movement patterns across different farming environments. These variations necessitate targeted optimization of pig counting algorithms for specific environmental conditions. This study proposes a dynamic pig counting algorithm, YOLOv8n-EGV+Deep SORT-P, which enhances the YOLOv8n+Deep SORT model. The YOLOv8n model serves as the detector, while the ELA attention mechanism is incorporated to improve detection accuracy for pig targets under challenging conditions such as occlusion, deformation, and poor lighting. A lightweight module is also incorporated to enhance detection speed without sacrificing precision. The Deep SORT tracking model is enhanced through improvements to the feature extraction network and the introduction of CIoU, effectively addressing tracking challenges such as target loss and identity switching while improving algorithm efficiency and stability. Finally, the virtual counting line method provides a straightforward and efficient approach to dynamic pig counting, performing optimally with the improved tracking algorithm. The main contributions of this study are as follows:

1. Development of a comprehensive pig detection and tracking dataset collected from corridor videos in real farming environments. Extensive experimental comparisons validate the effectiveness of the proposed improvements, ensuring the method’s reliability and practical applicability.
2. Introduction of the YOLOv8n-EGV algorithm for pig detection. The improved algorithm incorporates the ELA (Efficient Local Attention) mechanism and lightweight convolutional modules (GSConv and VOVGSCSP), which enhance detection accuracy and diminish computational and network structure complexity, resulting in markedly increased detection speed.
3. Development of the Deep SORT-P algorithm for pig tracking, which introduces an improved DenseNet-based feature extraction network and CIoU matching algorithm. These improvements augment the precision of multi-object tracking and tracking robustness under challenging farm conditions.
4. Implementation of a virtual counting line technique for monitoring pigs moving traversing farming corridor. Comparative analysis demonstrates that the improved YOLOv8n-EGV+Deep SORT-P model significantly outperforms the original model in counting accuracy.

## 2. Methods

**Figure 1:**
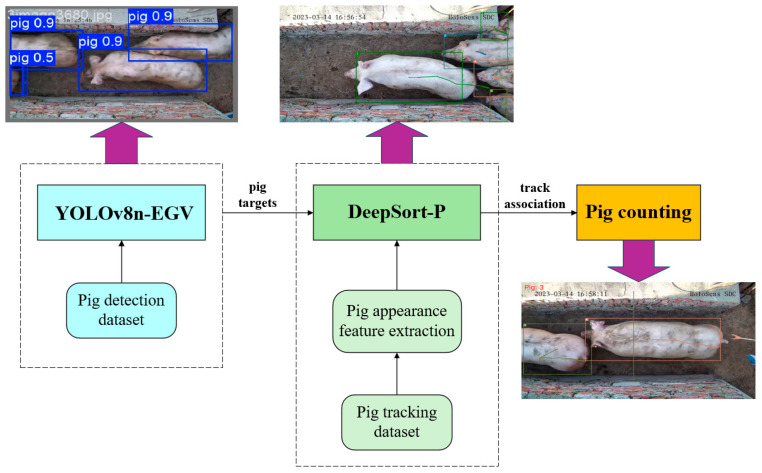
Comprehensive technology roadmap.

This study proposes an enhanced YOLOv8n-EGV+Deep SORT-P algorithm for high-precision dynamic pig counting. The algorithm comprises three main modules: the detection module, the tracking module, and the counting module. The overall technical roadmap of the algorithm is illustrated in Figure 1.

The proposed system operates in a sequential pipeline. First, the improved YOLOv8n-EGV algorithm detects pigs in each video frame and forwards the detection results to the improved Deep SORT-P pig object tracking algorithm. Upon receiving the pig detection results in the current image, the Deep SORT-P algorithm retrieves the detection results from the previous frame to predict pig movement trajectories in the current frame. Next, the pig re-identification network calculates the correlation between the predicted results of the previous frame and the current detection results. Finally, the virtual counting line method dynamically counts the pigs as they traverse the corridor.

### 2.1. Detector

#### 2.1.1. YOLOv8n

**Figure 2:**
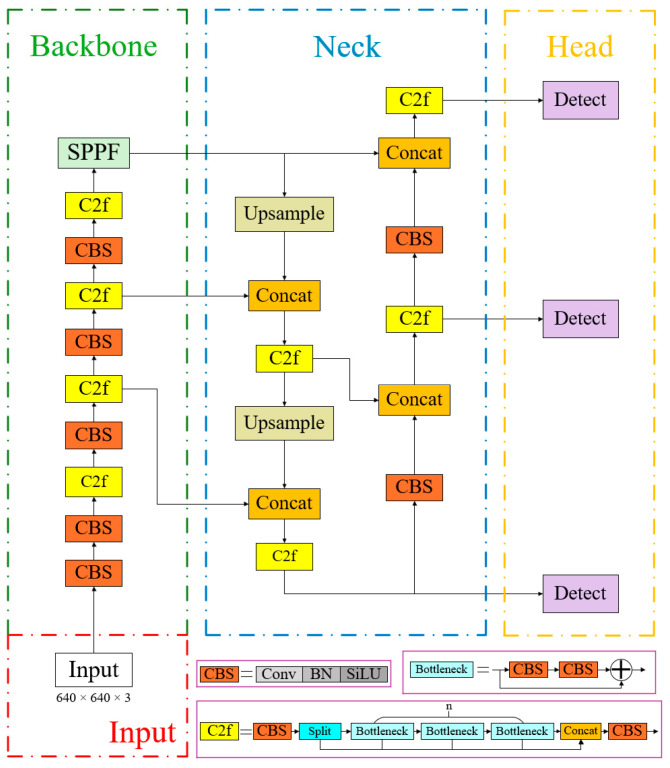
Structure of the YOLOv8n model.

The YOLO [30] (You Only Look Once) series represents a landmark family of classic single-stage object identification algorithm, excelling in both accuracy and speed of detection. In 2023, the Ultralytics team released the YOLOv8 algorithm, whose network architecture is shown in Figure 2. This algorithm builds upon the YOLOv1-v7 series models, reducing network parameters while improving both detection accuracy and real-time performance, leading to its widespread adoption across various object detection and segmentation applications.

YOLOv8′s backbone network adheres to the CSPDarkNet structure, comprising three key components: the Conv convolution module, the C2F module, and the SPPF module. The C2F module, inspired by the ELAN concept in YOLOv7, enhances the model’s gradient flow by integrating additional gradient flow branches. The SPPF module adaptively transforms feature maps of varying sizes into fixed-size outputs while efficiently integrating local and global feature information. The standard convolution block (CBS) in YOLOv8 consists of Conv2d, BatchNorm2d, and the Silu activation function. The neck component retains the bidirectional feature pyramid network structure (FPN+PAN) from YOLOv5, enabling multi-scale object prediction by merging features from different layers. Feature maps from the backbone network are processed through the Feature Pyramid Network (FPN) in a top-to-bottom manner, transmitting high-level semantic information. This process fails to convey feature localization information, whereas the bottom-up structure of the PAN (Path Aggregation Network) transmits low-level localization features upwards, enabling the network to perform multi-scale detection while effectively propagating both semantic and spatial information. YOLOv8′s head component represents a significant architectural advancement compared to its predecessors. The original merged head structure has been supplanted by the decoupled head structure (Decoupled-Head), which separates classification and detection tasks. One head handles classification, measured by binary cross-entropy loss, whereas the other head performs detection, measured by bounding box loss. This decoupled approach allows for more specialized optimization of each task.

Based on variations in network depth and width, the YOLOv8 algorithm is divided into five types: n, s, m, l, and x. Among them, YOLOv8n has the smallest number of model parameters and the lowest number of floating-point operations, resulting in faster inference speed and offering excellent usability and customizability. In this study, considering both detection accuracy and speed, YOLOv8n is selected as the baseline model.

#### 2.1.2. YOLOv8n-EGV

**Figure 3:**
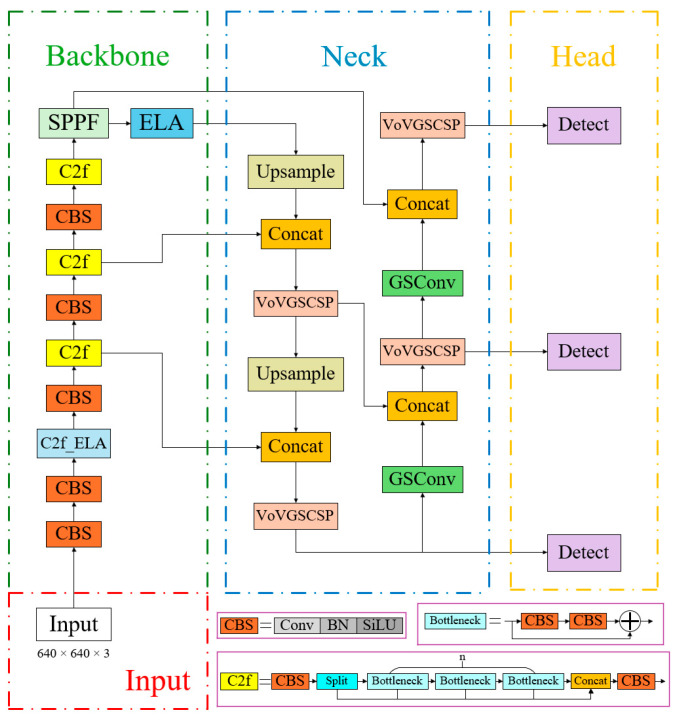
Structure of the YOLOv8n-EGV model.

To address the limitations of the standard YOLOv8n algorithm when detecting pigs under challenging conditions—such as occlusion, deformation, partial visibility, and poor lighting—this study proposes the YOLOv8n-EGV detection network. The new network incorporates an efficient local attention mechanism and a lightweight convolutional module, as illustrated in Figure 3. The YOLOv8n-EGV model features two key architectural improvements. Firstly, the Efficient Local Attention (ELA) mechanism is incorporated into the backbone network to enhance the representational capability of the CNN. The ELA structure is lightweight and straightforward, precisely identifying regions of interest while improving detection accuracy. Secondly, the Conv module in the Neck layer of the YOLOv8n network is substituted with GSConv, and the C2f module in the Neck is replaced with VoV-GSCSP. This modification reduces the model’s parameter count while maintaining detection accuracy, thereby rendering the model more lightweight. The specific improvement methods are outlined below:

##### ELA

**Figure 4:**
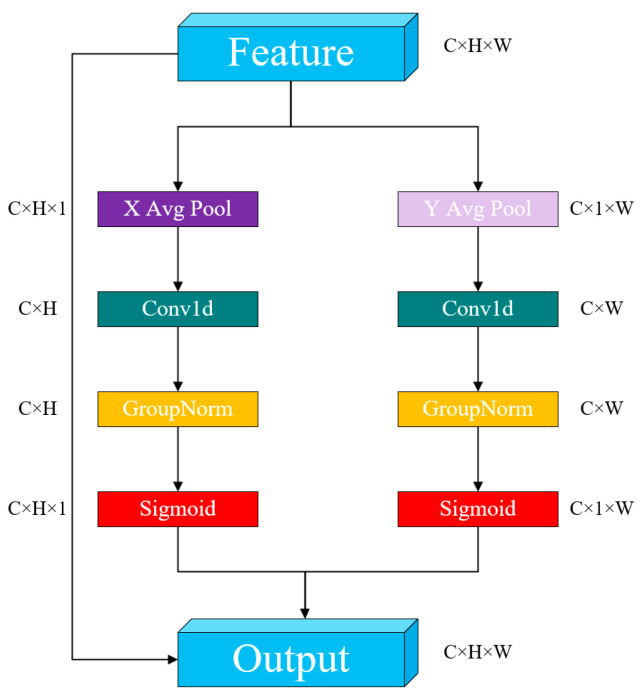
ELA algorithm structure.

ELA (Efficient Local Attention) [31] is an efficient local attention mechanism, illustrated in Figure 4. Despite its simple architecture, ELA significantly enhances detection performance by precisely identifying regions of interest while maintaining input feature map channel dimensions, and its lightweight characteristics. The ELA mechanism operates through the following process: First, similarly to CA, ELA utilizes strip pooling in the spatial dimension to obtain feature vectors in both horizontal and vertical orientations. It preserves a narrow kernel shape to capture long-range relationships and mitigate the influence of irrelevant regions on label prediction, thereby acquiring rich target location features in their respective matrices. Second, ELA processes these horizontal and vertical feature vectors independently using 1D convolutions for local interactions. The kernel size can be adjusted as needed to control the scope of these interactions. The resulting vectors undergo group normalization (GN) followed by nonlinear activation to generate directional attention predictions. The final position attention is obtained by multiplying the position attention from both directions. Compared to 2D convolutions, 1D convolutions are more adept at handling continuous signals, and are lighter and faster. In comparison to BN, GN demonstrates comparable performance and greater versatility.

Compared to some well-established attention mechanisms, such as SE [32] (Squeeze-and-Excitation) and CBAM [33] (Convolutional Block Attention Module), the ELA attention mechanism demonstrates superior performance. SE mainly focuses on adjusting the channel-wise weights, neglecting the crucial role of spatial information in occlusion and local structural changes. Although CBAM incorporates spatial attention, its spatial modeling is relatively shallow, making it difficult to capture detailed local features of the target adequately. The ELA attention mechanism strengthens the modeling of local feature regions and places greater emphasis on fine-grained information in key areas of the target. As a result, it can effectively extract discriminative regional features even when the target is partially occluded or deformed. ELA typically employs deformable or multi-scale receptive field designs, which enhance its spatial selectivity and dynamic adaptability, enabling it to accurately locate and identify targets in complex backgrounds. By enhancing the response to local textures and edge regions, ELA exhibits greater robustness to strongly structured local features, such as contours and boundaries. It maintains focus on key areas even under blurry visual conditions, such as low-light environments, thereby improving detection accuracy. Consequently, in the real-world scenarios of this study, ELA demonstrates superior performance compared to certain established attention mechanisms.

##### GSConv

**Figure 5:**
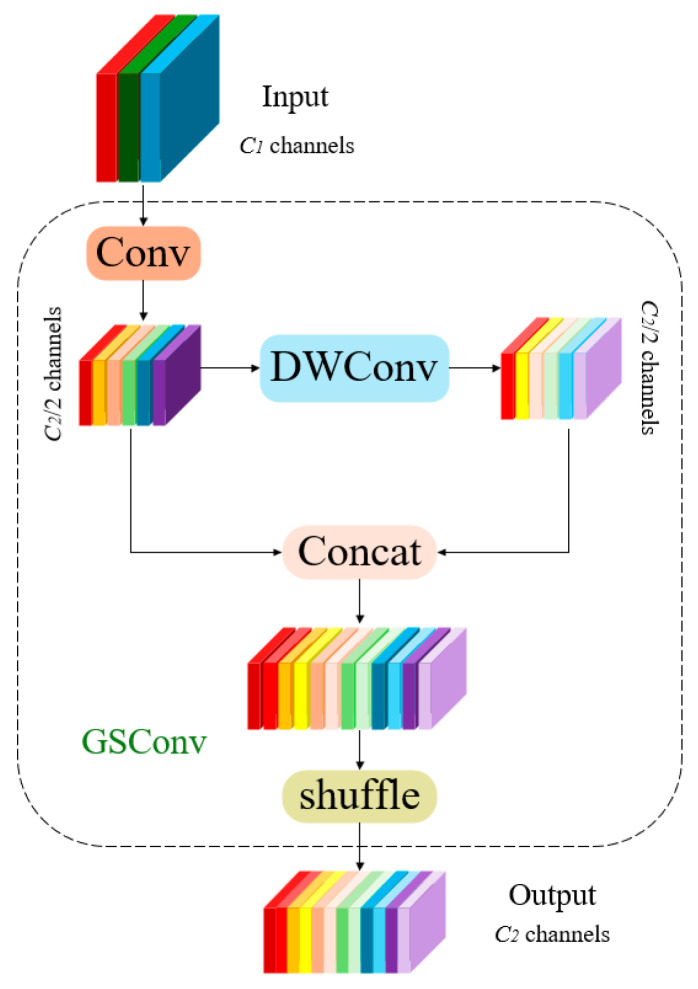
GSConv algorithm structure.

GSConv [34] is an innovative convolutional operation aimed at enhancing the performance and efficiency of deep learning models. Its core principle is based on the integration of group convolutions and a displacement mechanism [35]. First, GSConv segments the input feature map into multiple groups, performing convolution operations on each group independently. This method markedly decreases the parameter count and computational complexity while maintaining model expressiveness. Subsequently, during the convolution process, GSConv introduces a displacement operation that shifts certain channels of the feature map horizontally, enhancing the acquisition of local contextual information. After displacement, the feature map is reassembled by augmenting or concatenating elements to integrate information from different groups, allowing the model to merge information from multiple directions and further improve overall performance. GSConv is not only more computationally efficient than conventional fully connected convolutions, making it suitable for resource-constrained devices, but also enhances feature learning capability, enabling superior performance in managing intricate patterns. In convolutional neural network architectures, standard convolution operations yield more accuracy but result in increased model complexity and prolonged inference durations. Conversely, while DWConv (depth-wise separable convolution) provides expedited detection speed, its accuracy is diminished. Therefore, the design objective of the GSConv module is to diminish model complexity while preserving detection accuracy. The structure of the module is presented in Figure 5. GSConv integrates the semantic information generated by standard convolution into various parts of DWConv, fully leveraging the advantages of both standard and DWConv convolutions. This approach accelerates DWConv and enhances the accuracy of standard convolutions, thereby achieving better performance in the pig detection task.

To accelerate the computation of predictions, the feedforward images in CNNs almost always undergo analogous transformation processes in the Backbone, wherein spatial information is progressively transferred to the channel dimension. Moreover, each instance of compressing the spatial dimensions (width and height) of the feature map while expanding the channels results in the inevitable loss of semantic information. Dense convolution calculations maximize the retention of hidden connections between channels, while sparse convolutions completely sever these connections. GSConv preserves these connections as much as possible. Nevertheless, if employed at every stage of the model, the network would become excessively deep, and the increased depth would impede data flow, considerably prolonging the inference time. Upon reaching the Neck, these feature maps are lengthened (with the channel dimension maximized and the width and height dimensions minimized), rendering further alterations superfluous. Consequently, a more effective strategy is to implement GSConv only in the Neck layer. At this juncture, employing GSConv to process concatenated feature maps is preferable, as it reduces redundancy and repetition of information, eliminating the necessity for further compression, thus enhancing the efficacy of attention modules such as SPP and CA.

##### VOVGSCSP

**Figure 6:**
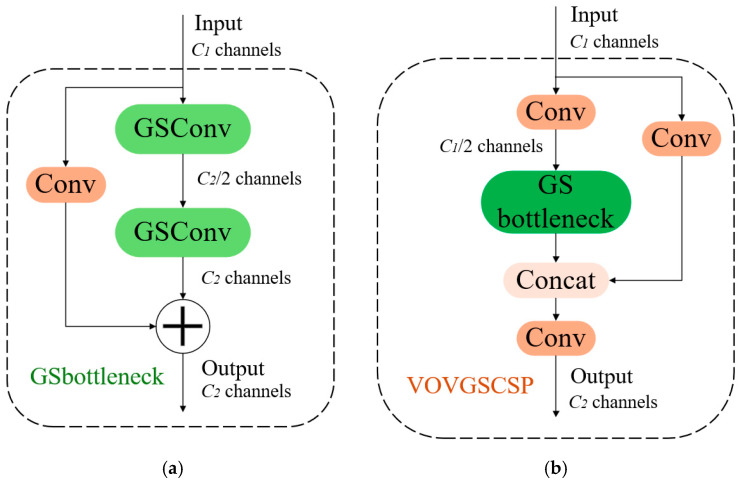
(**a**) Structure of the GSbottleneck model; (**b**) Structure of the VOVGSCSP algorithm.

The cross-level part of the VOVGSCSP [34] network is constructed utilizing a singular aggregation method. This approach effectively integrates information between feature maps from different stages. The stacking GSConv host convolutions further augment the GS bottleneck layer structure, improving the network’s feature processing capabilities, strengthening the nonlinear expression of the features, and increasing information reutilization. Although GSConv can markedly reduce redundant information in the feature maps of the pig detection model, it has limitations in further decreasing inference time without compromising accuracy. Consequently, the C2f module in the Neck network is replaced with the VoVGSCSP module. VoVGSCSP consists of GSConv modules aggregated by a singular technique, as shown in Figure 6a. The structure of VoVGSCSP is illustrated in Figure 6b. The implementation of the VoVGSCSP module decreases both computational complexity and network structure complexity while preserving detection accuracy. This further reduces the model’s memory usage, accelerates inference performance, and optimizes feature utilization, making the model more suitable for lightweight tasks.

### 2.2. Tracker

#### 2.2.1. Deep SORT

The SORT [36] (Simple Online Realtime Tracking) algorithm employs Kalman filtering and the Hungarian algorithm for multi-target tracking, providing the benefits of simplicity and high speed. Nevertheless, the SORT algorithm only predicts the tracking trajectory based on the motion trends of the target, disregarding the object’s appearance characteristics. The Kalman filter employs a linear constant-velocity model for prediction; however, in real-world tracking, the object’s velocity may fluctuate. Moreover, the SORT algorithm is exclusively designed for short-term target tracking and is ineffective in scenarios including target occlusion. These constraints result in frequent identity switches and low tracking accuracy in the presence of occlusion.

**Figure 7:**
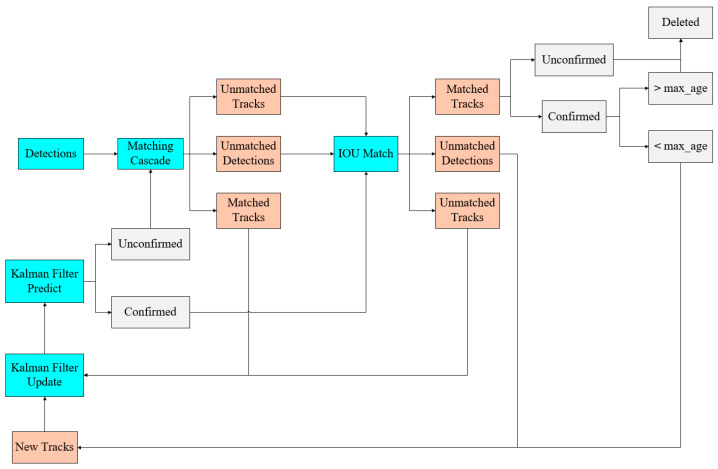
Deep SORT module structure.

To address the limitations of the SORT algorithm, Alex Bewley et al. proposed an enhanced tracking algorithm, Deep SORT [37] (Simple Online Realtime Tracking with Deep Association Metric). This improved approach incorporates appearance information, combining motion and appearance features with a linear weighted value as the ultimate measure for matching using the Hungarian algorithm. Additionally, Deep SORT employs a cascade matching strategy, assigning higher matching priority to objects that appear more frequently. The algorithm incorporates the extraction of deep learning features and similarity metrics, wherein appearance feature comparisons are made for all targets during each tracking process to reduce identity switches and achieve more sustained tracking. The network structure of the Deep SORT algorithm is shown in Figure 7. The Deep SORT architecture consists of two main components: the deep appearance descriptor branch and the motion prediction branch. The motion prediction branch uses Kalman filtering to predict the trajectory state, leveraging data from previous frames to project the target’s position in the subsequent frame. The Mahalanobis distance is employed to assess the difference between the predicted trajectory and the actual detection. The deep appearance descriptor branch operates as a convolutional network for image classification, extracting appearance features from the detected frames and converting them into feature vectors. Cosine distance metrics are utilized to evaluate the similarity between these feature vectors. The trajectory segments are subsequently linked using a cascade matching algorithm that combines both cosine and Mahalanobis distances. During the tracking management phase, the tracks are updated, initialized, and deleted to ensure the continuity of effective tracking.

#### 2.2.2. Deep SORT-P

To mitigate target loss and frequent identity switches during pig tracking with the Deep SORT algorithm, this study proposes a pig tracking algorithm, Deep SORT-P, which incorporates an efficient re-identification feature extraction network and an advanced IoU matching algorithm. The specific improvements are described as follows:

##### DenseNet

The original Deep SORT employs ResNet [38] as the re-identification model to extract target features, which effectively improves the target tracking performance and facilitates more precise identification and tracking of the target, thereby enhancing the overall tracking performance and stability. However, in this study, the pigs are monitored in a channel environment, where mutual occlusion and squeezing are particularly pronounced, especially when they first enter the channel. This creates a more complex scenario, requiring stronger re-identification capabilities. A re-identification network with enhanced feature extraction ability is employed to perform deeper feature extraction of the pigs’ appearance, aligning with the demands of the actual pig tracking process.

DenseNet [39] is a densely connected model that differs from ResNet, which incorporates the input and output directly to establish a residual structure. In DenseNet, the input and output are connected in parallel, with each layer linked to all preceding levels, receiving all the outputs of preceding layers as additional inputs. This allows each layer within the module to directly access the outputs of all prior layers, enabling the reuse of features. The network can extract and utilize more features from the input data, thereby enhancing its expressive power. This extensive use of features enables relatively simple network structures to achieve better performance. As shown in Formula (1), the input of the i-th layer is contingent not only upon the output of the (*i* − 1)-th layer but also on the outputs of all previous layers.

(1) $$X_{i}=H_{i}\left(\left[X_{0},X_{1},\ldots ,X_{i-1}\right]\right)$$

In this context, [] represents the concatenation operation, where all outputs from $X_{0}$ to $X_{i-1}$ are combined along the channel dimension. H denotes the nonlinear transformation, which is a combination of BN + ReLU + Conv (3 × 3).

During the target tracking process, when occlusion arises, one crucial method for Deep SORT to maintain consistent ID is the implementation of cascade matching between the appearance features derived from the re-identification network and the detection boxes. The matching results directly affect the total ID switch rate of the tracking model. Consequently, the DenseNet model, capable of extracting greater visual information, is more appropriate for the job of appearance feature extraction in the context of this study. This research employs DenseNet-121 as the feature extraction network to enhance pig re-identification performance, minimize frequent identity switches during tracking, and improve the tracking model’s accuracy. The structure of the DenseNet-121 network is illustrated in Table 1.

##### CIoU

In the second stage of the Deep SORT algorithm, while the IoU matching algorithm offers scale invariance, it exhibits limitations as an overlap metric due to the significant scale variations in pig movement and the presence of occlusions. In contrast, CIoU matching evaluates not only the overlap area but also the distance between center points and aspect ratio. As a result, CIoU matching can reduce trajectory matching errors and decrease the number of identity switches. The expression is as follows:

(2) $$CIoU=IoU-\left(\frac{\rho^{2}\left(b,b^{gt}\right)}{c^{2}}\right)+\alpha v$$

where *ρ* represents the Euclidean distance between the center points of the two detection boxes; *b* and *b^gt^* denote the center points of the predicted bounding box and the ground truth bounding box, respectively; *c* represents the diagonal length of the smallest enclosing box that encompasses both bounding boxes; α is a balancing factor that regulates the impact of the centroid distance and the bounding box size; *v* is a parameter utilized to assess the consistency of the aspect ratio between the predicted and ground truth bounding boxes. After integrating CIoU into the Deep SORT object tracking algorithm, the similarity between bounding boxes is evaluated from a more holistic standpoint. This is particularly beneficial in target tracking scenarios that involve occlusion, scale variation, or shape deformation. When monitoring a large number of targets with frequent occlusions, CIoU can more accurately measure the similarity between detection boxes, mitigating the impact of occlusions caused by pigs crowding together. This enhances the model’s representational capability and significantly improves the precision of the object tracking process.

### 2.3. Counting Technique

**Figure 8:**
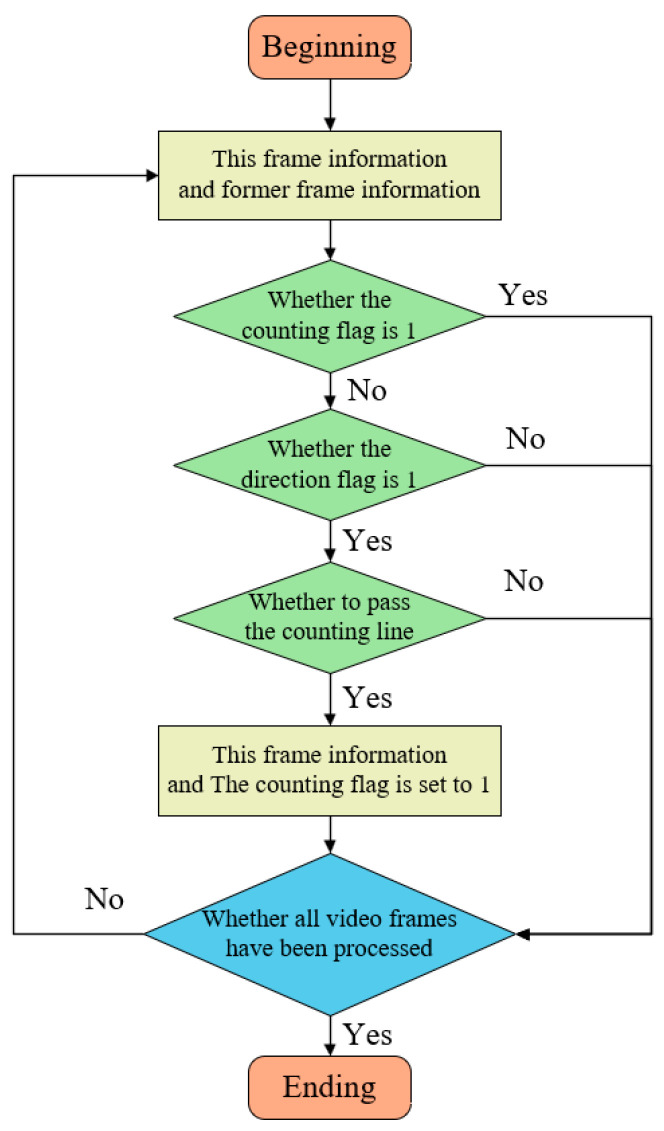
Flowchart of the counting methodology.

This study employs the virtual counting line method for object counting. This approach is straightforward and effective, and demonstrates commendable counting performance when the detection and tracking algorithms achieve high accuracy. The counting process is illustrated in Figure 8.

The key aspect of implementing this counting method in Deep SORT is the incorporation of the virtual counting line judgment into the multi-object tracking process. In this study, the counting line is manually defined by setting specific coordinates. A vertical counting line is positioned at the center of the screen, extending beyond the channel’s width to vertically span the entire channel. When a target crosses the counting line, logical conditions determine whether the counting is valid. During the tracking process in Deep SORT, each target is allocated a unique ID and trajectory. To ensure that a target is correctly counted upon crossing the counting line, a “counting flag” is designated for each target, indicating whether it has been previously counted. The counting flag is defined as a binary variable (0 or 1), with 0 signifying that the target has not been counted, and 1 indicating that it has been counted. In each frame, Deep SORT updates the state of each target. Upon a target crossing the counting line and meeting specific conditions, its trajectory’s counting flag is set to 1, and the count is incremented. If the counting proves unsuccessful, the system verifies if the “direction flag” of the target trajectory is configured to 1. The direction flag is a binary variable, where 1 represents movement to the left and 0 represents movement to the right. If the direction is erroneous, the trajectory is disregarded. If the direction is correct, the system further verifies whether the target’s trajectory has crossed the counting line. If so, the count is incremented, and the trajectory’s counting flag is set to 1; otherwise, the trajectory is discarded.

In summary, each time Deep SORT processes a frame, it initially updates the target’s position, velocity, and visual characteristics. Subsequently, for each target, it verifies if the counting flag is set to 1. If the flag is set, it indicates that the target has been previously counted, and the target is omitted. Next, if the target has not been counted, its direction of movement is examined. If the direction is incorrect, the target is skipped. If the target’s movement direction is correct, it is further verified whether it has crossed the counting line. Upon fulfillment of the condition, the count is augmented, and the target’s counting flag is set to 1. Finally, all counting results are aggregated, and upon processing all video frames, the total number of targets that have crossed the counting line is obtained.

## 3. Experiment and Discussion

### 3.1. Experimental Environment and Setup

Establishing an appropriate experimental environment is crucial for the successful execution of the experiment, and the process of constructing the experimental environment is of paramount importance. This study primarily focuses on the detection, tracking, and counting of pigs. The experiment was conducted under the conditions outlined in Table 2.

### 3.2. Pig Detection Experimental Results and Discussion

#### 3.2.1. Production of the Pig Detection Dataset

This study utilized data collected from a pig farming facility, comprising approximately 260 min of video footage of pigs. The video resolution is 1280 × 720, and the data were recorded on 14 March 2023, from 16:30 to 22:30. To validate the algorithm’s applicability, the experimental data were gathered from an actual passageway used in the farm. The passageway is 1.6 m wide, and all pigs traversed it at a standard velocity (v ≤ 2.5 m/s).

**Figure 9:**
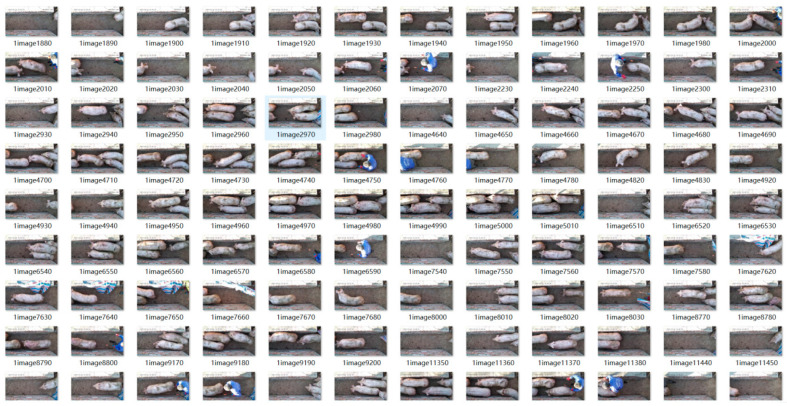
Pig target dataset.

Using a custom Python frame extraction, the video of pigs passing through the passageway was segmented into individual images. The frame extraction interval was set to 10 to avoid excessive similarity between frames. After filtering the extracted frames, images without targets and those with excessively high similarity were removed. A total of 1568 images were obtained and saved in .jpg format. Sample images from the pig dataset are shown in Figure 9.

**Figure 10:**
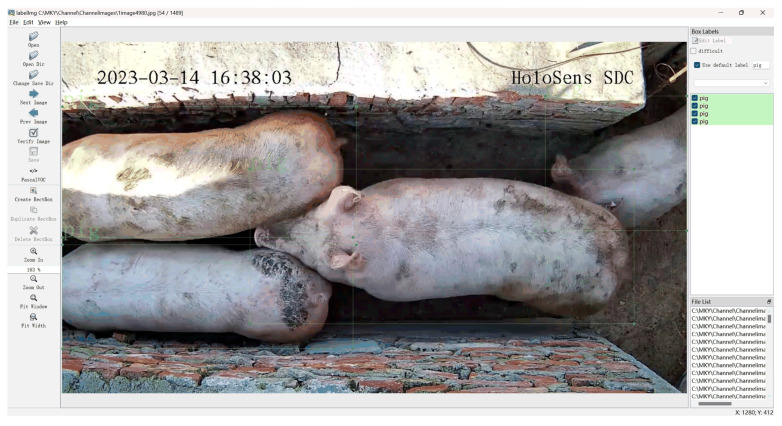
LabelImg annotation interface.

The open-source software labelImg 1.8.6 was used for manual annotation of pigs in the images. The annotation format was set to PASCAL VOC, with annotation files saved in “.xml” format and the label identified as “pig”. The annotation interface is illustrated in Figure 10. The pig detection dataset was further divided into training, testing, and validation sets in a 7:2:1 ratio, resulting in 1098 images for training, 314 images for testing, and 156 images for validation.

#### 3.2.2. Evaluation Indicators for Detection

This study employed precision (P), recall (R), and mean average precision (mAP) to evaluate the model’s effectiveness in pig object detection. Higher values of precision, recall, and mean average precision indicate a higher rate of accurate recognition and superior detection efficacy of the algorithm. The F1-score, which evaluates both precision and recall, is used to assess the model’s accuracy and completeness in detecting all targets. Additionally, model parameters (M) and total floating-point operations (FLOPs) were used to measure the complexity of the model. A smaller number of model parameters and lower FLOPs indicate a more lightweight model.

Precision (*P*) is a metric used to evaluate the correctness of the final predicted results for a specific class of objects. It represents the proportion of correctly detected pig targets to the total number of detected targets. The formula for precision is presented in Equation (3).

(3) $$P=\frac{TP}{TP+FP}$$

In the equation, *TP* (True Positives) represents the number of pigs that are accurately detected, while *FP* (False Positives) denotes the number of objects erroneously classified as pigs.

Recall (*R*) is a metric used to evaluate the capacity of an object detection model to detect all relevant targets. It represents the proportion of correctly identified pig targets to the total number of pigs that should be detected. The formula for recall is presented in Equation (4).

(4) $$R=\frac{TP}{TP+FN}$$

In the equation, *TP* (True Positives) represents the number of pigs accurately identified, while *FN* (False Negatives) denotes the number of pig targets that were missed.

Average Precision (*AP*) is defined as the area under the Precision–Recall (*PR*) curve. Its value is obtained by computing the integral of the function derived from precision and recall, as expressed in Equation (5).

(5) $$AP=\int_{0}^{1}P(R)dR$$

Mean Average Precision (mAP) represents the mean value of the average precision across all categories, and its formula is given in Equation (6). Since this experiment involves only a single category, namely pig targets, it is fundamentally a binary classification problem. Therefore, mAP is equivalent to AP, which corresponds to the area under the Precision–Recall (PR) curve.

(6) $$mAP=\frac{\sum_{i=1}^{c}AP(i)}{c}$$

The *F*1-*score* represents the harmonic mean of recall and precision, with values ranging from 0 to 1. The formula is presented in Equation (7).

(7) $$F1\;score=\frac{2\times precision\times recall}{precision+recall}$$

#### 3.2.3. Training and Discussion on Pig Detection Algorithms

**Figure 11:**
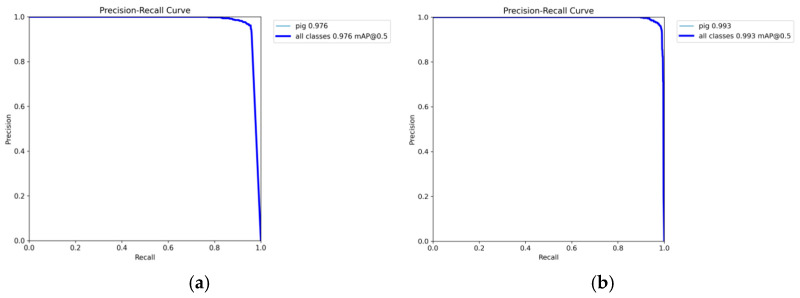
Outcomes of different algorithms for pig detection. (**a**) YOLOv8n algorithm; (**b**) YOLOv8n-EGV algorithm.

Figure 11a presents the Precision–Recall curve for pig object detection training using the YOLOv8n algorithm, whereas Figure 11b depicts the Precision–Recall curve for training with the YOLOv8n-EGV algorithm. A comparison of these two experimental curves demonstrates that the improved YOLOv8n-EGV model significantly outperforms the original YOLOv8n model.

With an IoU threshold of 0.5, the mAP increases from 0.976 to 0.993, representing a 1.7% improvement over the original model. In the YOLOv8n model, precision shows a slight decline at higher recall levels (Recall > 0.8). While the balance between precision and recall is effectively maintained, precision drops more significantly as recall approaches 1. In contrast, the improved YOLOv8n-EGV model demonstrates superior stability in precision at higher recall levels. Even when recall approaches 1, precision remains consistently high. The improved PR curve approaches the upper right corner of the graph, indicating that the enhanced model achieves significantly higher precision across different recall levels.

This study evaluates the effectiveness and progress of the enhanced algorithm by comparing the YOLOv8n-EGV object detection algorithm with several other models, including YOLOv5n, YOLOv7-tiny, YOLOv8n, YOLOv9n, and YOLOv10n. The findings are presented in Table 3, and analyzed using seven evaluation metrics: Params, FLOPs, Precision (P), Recall (R), mean Average Precision (mAP), F1-score, and Frames Per Second (FPS). The improved YOLOv8n-EGV model achieves the highest mAP and F1-score, signifying its superior detection accuracy on the dataset used in this research. Compared to the baseline YOLOv8n model, the mAP increases by 1.7%, and the F1-score improves by 2%. Additionally, the number of parameters (Params) and the floating-point operations (GFLOPs) are reduced by 14.3% and 17.3%, respectively, leading to a significant improvement in detection speed. The results demonstrate that, compared to conventional detection models, the YOLOv8n-EGV model offers certain advantages in overall performance.

#### 3.2.4. Results and Discussion on Pig Detection

**Figure 12:**
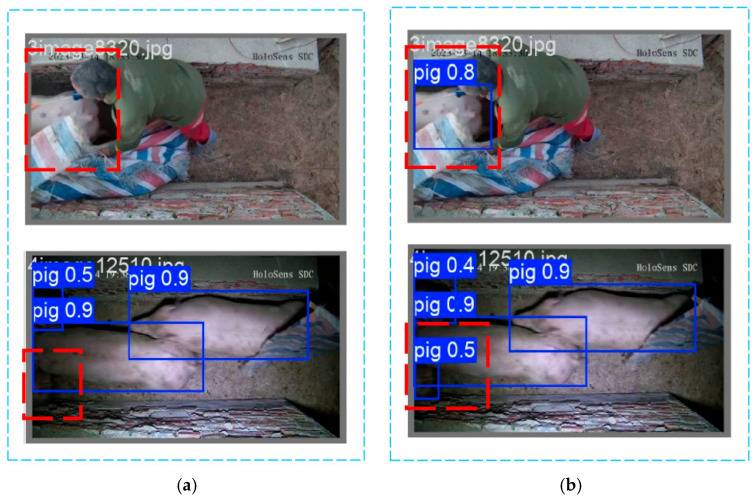
Comparison with different detection model results. (**a**) Detection results of YOLOv8n; (**b**) Detection results of YOLOv8n-EGV.

In Figure 12, panel a illustrates the pig detection results using the baseline YOLOv8n model, the undetected pig targets are highlighted using red dashed boxes. While panel b shows the detection results using the improved YOLOv8n-EGV model, the correctly detected pig targets and their corresponding detection bounding boxes are also highlighted using red dashed boxes. Under partial occlusion and low-light conditions, the improved model demonstrates superior capability in identifying local body parts of pigs compared to the baseline model. It accurately localizes target pigs by detecting partial body features, successfully annotating the targets, and providing explicit confidence scores. Consequently, the improved YOLOv8n-EGV model significantly enhances detection accuracy and reliability in complex situations, effectively reducing both missed detections and false positives. Furthermore, when used for future tracking and counting tasks, the improved detector model provides more precise initial target positions and bounding boxes, facilitating feature extraction and matching. Even when targets experience partial occlusion or shape distortion, the model maintains continuous target recognition, thereby improving the robustness of the tracking model.

#### 3.2.5. Ablation Experiment for Pig Detection

To evaluate the superior performance of the ELA attention mechanism under complex conditions, this study conducts comparative experiments by incorporating two widely used attention mechanisms, namely CBAM and SE, in addition to the proposed ELA mechanism. The experimental results are analyzed using four standard evaluation metrics: Precision (P), Recall (R), mean Average Precision (mAP), and F1-score, as shown in Table 4. When the CBAM attention mechanism is applied, Precision, Recall, and mAP increase by 0.5%, 1.6%, and 0.8%, respectively, while the F1-score remains unchanged. In contrast, the use of the SE attention mechanism results in a noticeably weaker improvement in detection performance compared to CBAM. Specifically, Recall and mAP increase by only 0.8% and 0.3%, respectively, with no change in Precision or F1-score. Incorporating the ELA attention mechanism yields substantial improvements across all four metrics. The Precision, Recall, mAP, and F1-score increase significantly by 0.9%, 3.1%, 1.5%, and 1.0%, respectively, demonstrating the enhanced detection performance of the proposed approach. These experimental results indicate that, under challenging conditions such as partial occlusion, deformation, and low illumination, the ELA attention mechanism outperforms other established attention mechanisms and is more effective in addressing the complex problems encountered in this study.

Table 5 outlines the modifications made to the pig detection network model to assess the efficacy of each enhanced module, analyzing four key metrics: Params, FLOPs, mAP, and F1-score. The comparison analysis indicates that the introduction of only the ELA attention mechanism module results in a significant increase in the mAP and F1-score by 1.5% and 1%, respectively. While the improved model with ELA achieves excellent accuracy, it also leads to an increase in model Params and FLOPs, reaching 3.14M and 8.3G, respectively. Incorporating the GSConv module along with the ELA attention mechanism reduces Params and FLOPs to 2.76 M and 7.2 G, respectively, while the F1-score further improves by 1%, and mAP remains unchanged. Upon the integration of the ELA attention mechanism and the VOVGSCSP module, Params and FLOPs decrease even more significantly to 2.69 M and 6.9 G, with mAP increasing by 0.2%. However, the F1-score remains unchanged, though it decreases by 1% compared to the model using the GSConv module. When the three modules (ELA, GSConv, and VOVGSCSP) are integrated simultaneously, the model achieves comprehensive improvements over the original YOLOv8n, with Params and FLOPs reduced to 2.58 M and 6.7 G, respectively, while the mAP and F1-score increase by 1.7% and 2%, respectively. Overall, the analysis indicates that the incorporated and substituted modules effectively enhance detection accuracy while reducing the model’s weight, ultimately improving both detection precision and processing speed in the final optimized model.

### 3.3. Pig Tracking Experimental Results and Discussion

#### 3.3.1. Production of the Pig Tracking Dataset

**Figure 13:**
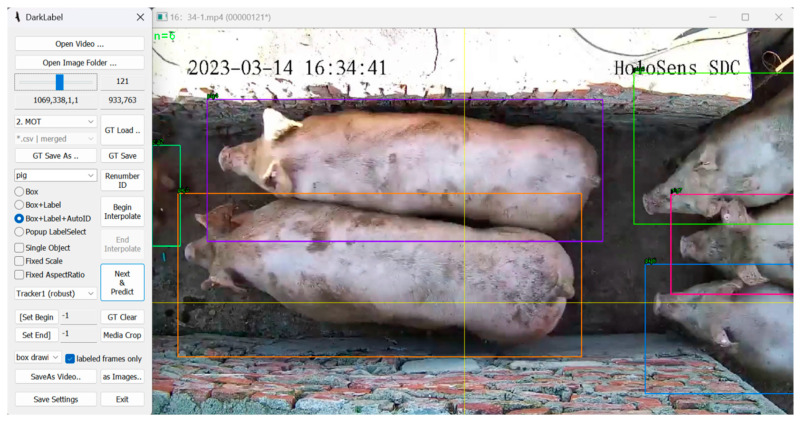
DarkLabel annotation interface.

A dataset for pig tracking was created by analyzing the collected video footage. The videos were segmented to remove portions without pigs. The target tracking dataset was then generated using the data annotation software DarkLabel 2.4. A total of 63 video segments were annotated, as shown in the annotation interface in Figure 13. The target category was set as “pig”, and the annotation information included trajectory IDs.

#### 3.3.2. Tracking Evaluation Indicators

Standard evaluation metrics for object tracking include IDSW, IDF1, MOTA, MOTP, and FPS.

IDSW (ID switches) refers to the total number of times the identity assignment of a single target changes during the tracking process. It is primarily used to evaluate the performance of a tracking algorithm in maintaining identity consistency for individual targets. An increase in IDSW occurs when the algorithm mistakenly reassigns a new ID to the same target or incorrectly assigns the same ID to different targets. A lower IDSW value indicates that the algorithm is more stable and reliable in maintaining consistent identities, while a higher IDSW value suggests greater errors, especially in challenging scenarios such as occlusion, target overlap, or rapid motion. Therefore, IDSW serves as a crucial metric for assessing the overall performance of multi-object tracking algorithms.

IDF1 represents the F1-score for identification, as computed by Equation (8). IDTP denotes the total number of targets accurately tracked with consistent IDs, IDFP denotes the total number of targets incorrectly tracked with consistent IDs, and IDFN denotes the total number of targets that were lost while tracking with consistent IDs.

(8) $$IDF1=\frac{2\times IDTP}{2\times IDTP+IDFP+IDFN}$$

MOTA (Multiple Object Tracking Accuracy) is one of the key metrics for evaluating the overall performance of an object tracking algorithm. It combines all major types of errors, including False Negatives (FN), False Positives (FP), and Identity Switches (IDSW), to assess the accuracy of object tracking. MOTA primarily focuses on the overall tracking accuracy, with its value typically ranging from 0 to 1. A higher MOTA value indicates better performance in reducing tracking errors. The calculation formula is presented in Equation (9).

(9) $$MOTA=1-\frac{\sum_{t}\left({FN}_{t}+{FP}_{t}+{IDSW}_{t}\right)}{\sum_{t}\left({GT}_{t}\right)}$$

where ${FN}_{t}$ signifies the count of targets that were not accurately detected by the tracker, ${FP}_{t}$ denotes the quantity of erroneously recognized targets, ${GT}_{t}$ refers to the number of ground truth bounding boxes, and ${IDSW}_{t}$ reflects the accuracy of the tracking algorithm.

MOTP (Multiple Object Tracking Precision) is a key metric used to measure the positional alignment between detected bounding boxes and ground truth boxes in object tracking algorithms. It primarily quantifies the localization accuracy of the detector, reflecting the spatial precision of the tracking algorithm. MOTP evaluates the alignment between detected and ground truth boxes by calculating the average error of all correctly matched targets. A higher MOTP value indicates a smaller matching error between the detected and ground truth boxes, suggesting that the tracking algorithm has high localization accuracy. Conversely, a lower MOTP value indicates a larger deviation between the detected and ground truth boxes, which may imply that the tracking algorithm has some error in target position estimation. The formula for calculation is presented in Equation (10).

(10) $$MOTP=\frac{\sum_{i,t}d_{t}^{i}}{\sum_{t}C_{t}}$$

where $d_{t}^{i}$ represents the overlap between the *i*-th detected bounding box and its corresponding ground truth bounding box in frame *t*, and $C_{t}$ indicates the number of successfully matched detected bounding boxes in frame t.

#### 3.3.3. Training and Discussion on the Pig Tracking Algorithm

In the private pig tracking dataset, the performance of the improved Deep SORT-P algorithm was compared with SORT, ByteTrack, and Deep SORT, as illustrated in Table 6. The Deep SORT-P algorithm demonstrated superior performance across the evaluation metrics MOTA, MOTP, IDF1, and IDSW, achieving 89.6%, 90.4%, 92.8%, and 79, respectively. The updated Deep SORT-P algorithm demonstrated a 4.2% improvement in MOTA relative to the previous Deep SORT algorithm, indicating better overall tracking accuracy and enhanced capability to manage occlusion and object loss. The MOTP increased by 1.7%, demonstrating enhanced precision in target localization and effectively reducing positional deviation. The IDF1 increased by 2.6%, reflecting more stable identity tracking and a significant reduction in identity mismatches. The IDSW declined from 106 to 79, representing an approximately 25.5% reduction, which effectively minimized identity switching and improved target continuity and consistency. However, the FPS fell from 63 to 59, indicating a minor decline in processing speed. Nevertheless, the algorithm maintained a relatively high frame rate, ensuring real-time applicability. Although the improved Deep SORT-P algorithm marginally compromises processing speed, its significant enhancement in tracking performance facilitates more stable and reliable pig tracking in complex environments.

#### 3.3.4. Pig Tracking Results and Discussion

**Figure 14:**
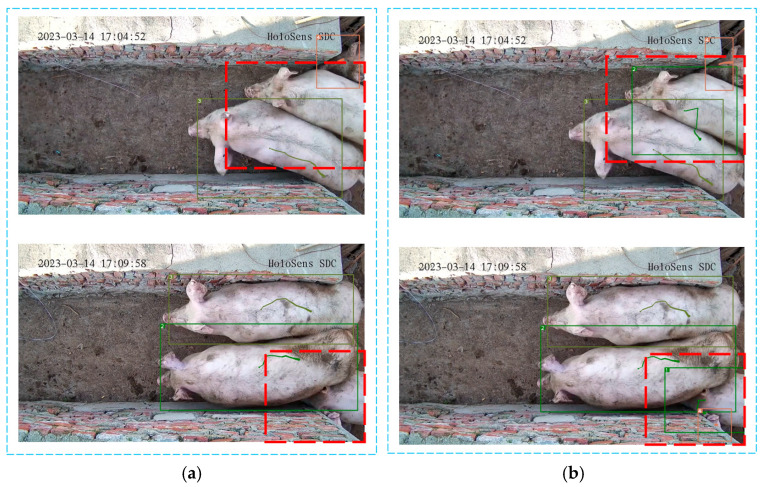
Comparison of target loss in pig tracking. (**a**) Tracking results of Deep SORT; (**b**) Tracking results of Deep SORT-P. The red dashed boxes in the figure represents the target tracking results that require special attention.

**Figure 15:**
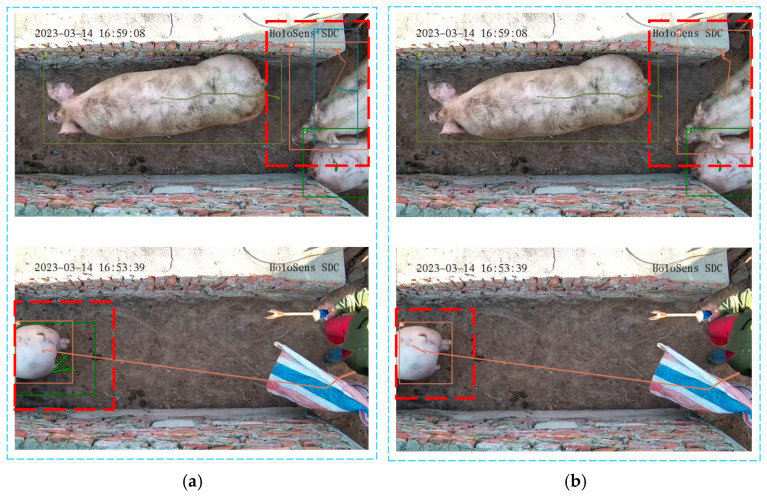
Comparison of false-positive tracking trajectories in pig tracking. (**a**) Tracking results of Deep SORT; (**b**) Tracking results of Deep SORT-P. The red dashed boxes in the figure represents the target tracking results that require special attention.

Figure 14 and Figure 15 compare the experimental outcomes of the Deep SORT algorithm with those of the improved Deep SORT-P algorithm. In Figure 14a,b, the red dashed boxes highlight instances where the original algorithm faces difficulties in tracking pigs during crowding or occlusions caused by narrow pathways. In these situations, the original algorithm experiences target loss during tracking. In contrast, the improved Deep SORT-P algorithm maintains stable bounding boxes even under the same occlusion conditions. This demonstrates a significant improvement in tracking continuity, with a notably reduced probability of target loss.

In Figure 15a,b, the areas enclosed by red dashed boxes indicate that the Deep SORT algorithm exhibits significant identity switch errors during pig tracking. In contrast, the enhanced Deep SORT-P algorithm demonstrates superior stability in pig tracking, significantly reducing the frequency of false-positive tracking trajectories. The consistency and stability of pig tracking targets are notably improved.

#### 3.3.5. Pig Tracking Ablation Experiment

Table 7 presents the modifications applied to the pig tracking model and analyzes four key performance metrics: MOTA, MOTP, IDF1, and IDSW, to evaluate the efficacy of the enhanced modules. Comparative analysis indicates that the introduction of the DenseNet-based re-identification feature extraction network results in increases of 3.3% in MOTA, 0.9% in MOTP, and 1.4% in IDF1, whereas IDSW is significantly reduced by approximately 18.9%. In contrast to solely implementing the DenseNet feature extraction network, the exclusive integration of the CIoU matching algorithm results in increases in MOTA, MOTP, and IDF1 by 1.4%, 0.2%, and 0.5%, respectively, with IDSW reduced by approximately 11.3%. Experimental results indicate that performance improvement of the pig tracking network, achieved by introducing only the CIoU matching algorithm, is considerably smaller than that of the DenseNet-based re-identification feature extraction network. The integration of both modules significantly enhances overall performance compared to the original Deep SORT, with MOTA, MOTP, and IDF1 rising to 89.6%, 90.4%, and 92.8%, respectively, while IDSW decreases to 79. The overall analysis suggests that the introduced and modified modules provide more reliable advantages in terms of tracking accuracy and stability.

### 3.4. Experiment and Discussion on Pig Counting

**Figure 16:**
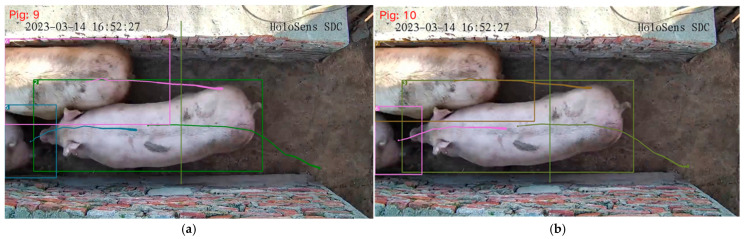
Results of different algorithms for pig counting. (**a**) YOLOv8n+Deep SORT counting algorithm results, the number is 9; (**b**) YOLOv8n-EGV+Deep SORT-P counting algorithm results, the number is 10.

To validate the counting accuracy of the final algorithm, this study conducted experiments using both the original YOLOv8n+Deep SORT counting algorithm and the improved YOLOv8n-EGV+Deep SORT-P counting algorithm. Figure 16a shows the counting results of the original algorithm, while Figure 16b illustrates the results of the enhanced algorithm. By manually counting the pigs in the video segment, it was determined that the precise count of pigs across the counting line was 10. The results indicate that the improved YOLOv8n-EGV+Deep SORT-P counting algorithm provided accurate results, whereas the original algorithm failed to count one target. This discrepancy arose from target loss and tracking interruption during the counting process, leading to counting errors.

Table 8 presents the experimental findings of tracking and counting pigs in 63 video segments captured from the designated passage. The original YOLOv8n+Deep SORT counting algorithm accurately counted 47 video segments, achieving an accuracy rate of approximately 74.6%. In contrast, the improved YOLOv8n-EGV+Deep SORT-P counting algorithm precisely enumerated 58 video segments, with an accuracy rate of approximately 92.1%. The experimental results indicate that the enhanced counting algorithm exhibits superior performance.

## 4. Conclusions

This study proposes an enhanced dynamic pig counting algorithm, YOLOv8n-EGV+Deep SORT-P. Compared to the original model, the improved algorithm enhances detection accuracy and speed in pig detection, particularly under occlusion scenarios such as crowding and deformation, as well as in low-light conditions. Additionally, it improves the precision and stability of multi-object pig tracking, mitigating issues such as target loss and frequent identity switches. Experimental results show that the mAP increased by 1.7%, while the Params and GFLOPs were reduced by 14.3% and 17.3%, respectively. Moreover, MOTA improved by 4.2%, MOTP increased by 1.7%, and IDSW decreased by approximately 25.5%. Finally, a virtual counting line method was employed to perform counting experiments on 63 video segments of pigs passing through a farm passage. When comparing the counting results before and after the algorithm enhancement, the improved algorithm achieved a counting accuracy of approximately 92.1%, representing a 17.5% increase. In summary, the proposed improved algorithm demonstrates significant superiority.

This study’s analysis indicates that future work can focus on the following directions:

1. The dataset established in this study consists of pigs of similar size and a single category. Future research could expand the dataset to include different pig categories, classified by size and color. The proposed improved algorithm can be adapted for the separate counting of various pig types, thereby broadening its applicability.
2. Further optimization and enhancement of the tracking model could be explored to improve counting accuracy and stability in more complex environments, ensuring a more comprehensive evaluation of the model’s effectiveness.
3. The counting strategy can be further optimized. This study utilizes a dataset of pigs being manually herded through the farm passage, eliminating the issue of duplicate counts due to pigs crossing back over the virtual counting line. Future work could introduce a bidirectional counting method, where pigs crossing the counting line in both the left and right directions are counted separately, and the final count is obtained by subtracting these two values. This enhanced counting strategy would improve the method’s application and effectively address issues when pigs traverse the route without human supervision, preventing duplicate counting.

## Figures and Tables

**Table 1:** DenseNet-121 network structure.

Layers	DenseNet-121	Output Size
Convolution	7 × 7 Conv, stride2	112 × 112
Pooling	3 × 3 Max pool, stride2	56 × 56
Dense Block I	$\left[\begin{array}{c} 1\times 1Conv\\ 3\times 3Conv \end{array}\right]\times 6$	56 × 56
Transition Layer I	$\left[\begin{array}{c} 1\times 1Conv\\ 2\times 2\;average\;pool,stride2 \end{array}\right]$	28 × 28
Dense Block II	$\left[\begin{array}{c} 1\times 1Conv\\ 3\times 3Conv \end{array}\right]\times 12$	28 × 28
Transition Layer II	$\left[\begin{array}{c} 1\times 1Conv\\ 2\times 2\;average\;pool,stride2 \end{array}\right]$	14 × 14
Dense Block III	$\left[\begin{array}{c} 1\times 1Conv\\ 3\times 3Conv \end{array}\right]\times 24$	14 × 14
Transition Layer III	$\left[\begin{array}{c} 1\times 1Conv\\ 2\times 2\;average\;pool,stride2 \end{array}\right]$	7 × 7
Dense Block IV	$\left[\begin{array}{c} 1\times 1Conv\\ 3\times 3Conv \end{array}\right]\times 16$	7 × 7
Classification Layer	$\left[\begin{array}{c} 7\times 7gllobal\;pool\\ 1000DFully-connected,softmax \end{array}\right]$	1 × 1

**Table 2:** Experimental environment.

Parameters	Configuration
System environment	Windows 11
CPU	Intel(R) Core(TM) i9-13900HX (Intel, Santa Clara, CA, USA)
GPU	NVIDIA GeForceRTX4060
Deep learning framework	Pytorch1.12
CUDA	11.3
Python	3.9

**Table 3:** Comparison of different models.

Model	Params/M	FLOPs/G	P/%	R/%	mAP/%	F1-Score/%	FPS
YOLOv5n	3.28	7.2	94.5	91.5	93.6	93	53
YOLOv7-tiny	6.2	6.56	96.1	93.8	95.8	94	49
YOLOv8n	3.01	8.1	98.2	95.7	97.6	96	65
YOLOv9n	4.1	11.5	98.4	93.2	96.8	95	61
YOLOv10n	2.96	8.4	97.8	93.6	95.4	94	58
YOLOv8n-EGV	2.58	6.7	99.2	98.8	99.3	98	72

**Table 4:** Pig detection experiment results with different attention mechanisms.

YOLOv8n	CBAM	SE	ELA	P/%	R/%	mAP/%	F1-Score/%
√				98.2	95.7	97.6	96
√	√			98.7	97.3	98.4	96
√		√		98.2	96.5	97.9	96
√			√	99.1	98.8	99.1	97

**Table 5:** Pig detection ablation experiment results.

YOLOv8n	ELA	GSConv	VOVGSCSP	Params/M	FLOPs/G	mAP/%	F1-Score/%
√				3.01	8.1	97.6	96
√	√			3.14	8.3	99.1	97
√	√	√		2.76	7.2	99.1	98
√	√		√	2.69	6.9	99.3	97
√	√	√	√	2.58	6.7	99.3	98

**Table 6:** Comparison of tracking results with other mainstream methods. The “↑” indicates that a higher value is better, while “↓” indicates that a lower value is preferable.

Tracker	Detector	MOTA↑	MOTP↑	IDF1↑	IDSW↓	FPS
SORT	YOLOv8n-EGV	75.9%	78.3%	76.6%	204	98
ByteTrack	YOLOv8n-EGV	83.7%	89.3%	88.4%	138	70
Deep SORT	YOLOv8n-EGV	85.4%	88.7%	90.2%	106	63
Deep SORT-P	YOLOv8n-EGV	89.6%	90.4%	92.8%	79	59

**Table 7:** Pig tracking ablation experiment results.

Deep SORT	DenseNet	CIoU	Detector	MOTA	MOTP	IDF1	IDSW
√			YOLOv8n-EGV	85.4%	88.7%	90.2%	106
√	√		YOLOv8n-EGV	88.7%	89.6%	91.6%	86
√		√	YOLOv8n-EGV	86.8%	88.9%	90.7%	94
√	√	√	YOLOv8n-EGV	89.6%	90.4%	92.8%	79

**Table 8:** Comparison results of different algorithms.

Model	YOLOv8n+Deep SORT	YOLOv8n-EGV+Deep SORT-P
Videos	63	63
Correct Counting Videos	47	58

## Data Availability

Data will be accessible upon request.
